# Fabrication of silicon films from patterned protruded seeds

**DOI:** 10.1063/1.4983575

**Published:** 2017-05-12

**Authors:** Huang Zeng, Wei Zhang, Jizhou Li, Cong Wang, Hui Yang, Yigang Chen, Xiaoyuan Chen, Dongfang Liu

**Affiliations:** 1 Department of Materials Science and Engineering, Shanghai University, 99 Shangda Road, Shanghai 200444, China; 2 Shanghai Advanced Research Institute, Chinese Academy of Sciences, 99 Haike Road, Shanghai 201210, China

## Abstract

Thin, flexible silicon crystals are starting up applications such as light-weighted flexible solar cells, SOI, flexible IC chips, 3D ICs imagers and 3D CMOS imagers on the demand of high performance with low cost. Kerfless wafering technology by direct conversion of source gases into mono-crystalline wafers on reusable substrates is highly cost-effective and feedstock-effective route to cheap wafers with the thickness down to several microns. Here we show a prototype for direct conversion of silicon source gases to wafers by using the substrate with protruded seeds. A reliable and controllable method of wafer-scaled preparation of protruded seed patterns has been developed by filling liquid wax into a rod array as the mask for the selective removal of oxide layer on the rod head. Selectively epitaxial growth is performed on the protruded seeds, and the voidless film is formed by the merging of neighboring seeds through growing. And structured hollows are formed between the grown film and the substrate, which would offer the transferability of the grown film and the reusability of the protruded seeds.

## I. INTRODUCTION

Silicon is the dominant semiconductor material in the modern electronic and photovoltaic industries. It is mostly used in the form of wafers sliced by costly wire-sawing from ingots.^1–5^ However, due to kerf loss, the slicing of ingot into wafers wastes great amount of expensive, high pure feedstock. Additionally, ultrathin wafers are hardly manufactured by wire-sawing because of concern about mechanical stability when sawn.^5^

In recent years, a direct conversion of feedstock gas to wafers technology based on epitaxial growth on porous silicon has been well developed.^6–15^ In this technology, a porous silicon bilayer was built by anodization of traditional Si mono-crystal wafers in HF solution with platinum as cathode; the pores on the surface were sealed by hydrogen annealing, forming a thin quasi-mono-crystal template for subsequent epitaxial growth of silicon; the unsealed porous layer was used as a sacrificial layer when the grown wafer was lifted off; and the thickness of grown wafer was controlled just by growth duration, available from several microns to hundreds of microns. And multiple use of the mother substrate can be achieved by rebuilding the porous bilayer after the grown wafer detached. Therefore, this gas-to-wafer technology provided a greatly raw-material-effective and cost-effective route to wafers by skipping the traditional Siemens’ poly-silicon synthesis, ingoting, and wire-sawing steps, and facilitated to realize flexible devices with the ultrathin wafers. Nowadays, thin flexible silicon crystals are spurring applications such as high efficient light-weight and flexible solar cells,^8–11,16,17^ SOI,^12^ flexible IC chips,^13^ monolithic 3D ICs and CMOS imager,^14,15^ and flexible transistors.^18^ However, it is rather challenging for the epitaxial technology based on porous silicon layers to avoid metallic contaminants from the electrolyte,^8^ and the reuse of mother substrates is limited by the re-creating step of the porous double layer in electrolyte solution.^19,20^

In this work, we demonstrate a prototype using patterned protruded seeds to epitaxially grow silicon films,^21^ intending to form uniformly hollows between the grown film and the mother substrate by the seed supporting structures, in this way, one approach might be achieved that the grown film / wafer could be lifted off without any sacrificial layer and the seeded mother substrate could be reused without any rebuilding steps in liquid.

## II. EXPERIMENTAL PROCESS

### A. The strategy of the prototype

**FIG. 1.**
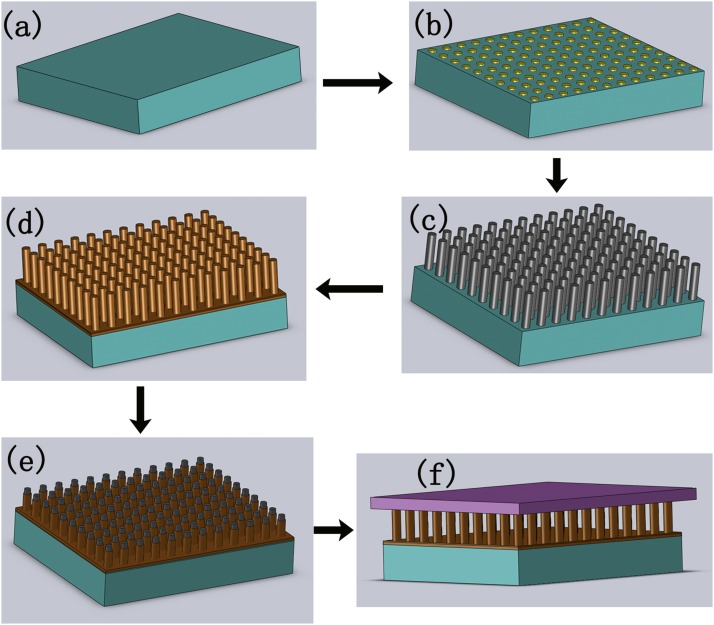
Schematic strategy of the prototype. a) Cz-Si wafer, i.e., the mother substrate. b) Photo-resist patterning the mother substrate by traditional photolithography. c) Fabrication of rod array by ICP dry etch. d) Preparation of oxide layer by thermal oxidization. e) Selective removal of oxide layer on the rod heads, forming patterned protruded seeds. f) Selective epitaxial growth of silicon film on the seeds.

The strategy of the prototype is schematically shown in Figure 1, a periodically patterned silicon rod array is prepared by the traditional photolithography and inductive coupled plasma (ICP) dry etching method using Czochralski mono-crystal silicon (Cz-Si) wafers as mother substrates; thermal oxidization is performed to form SiO_2_ layer on the surface of rods and the mother substrate, and this oxide layer will be used as a mask for later selectively epitaxial film growth; the oxide layer is selectively removed on the rod head, exposing the inner silicon core to form protruded seed; subsequently, selectively epitaxial growth is performed using the exposed silicon cores as seeds in a chemical vapor deposition system; As the growth evolved, the seeds grow larger and merge into a continuous film leaving uniformly structured hollows between the mother substrate and the grown film.

The formation of the structured hollows is the most important goal of our prototype, which, on one hand, makes the grown film in weak mechanical connection with the mother substrate only by the rods, thus will facilitate to mechanically lift off the grown film; on the other hand, can provide a passage-way for gases, liquids to run into under the grown film, allowing to perform such processing as etching and coating under the film. Furthermore, another goal of our prototype is that the uniformly distributed rods can give a stable mechanical support when devices are processed while the film still stands on the mother substrate, especially when the film is ultrathin.

### B. Fabrication of protruded seed patterns

4″, n-type, (100) oriented, 500 μm thick commercial Cz-Si wafers were used as the mother substrates. Traditional UV-photolithography was used to form photo-resist mask pattern for inductively coupled dry etching silicon to fabricate rod array; the rods had the diameter of 4μm and the length of 10-15μm, and the array had the period of 10μm.

**FIG. 2.**
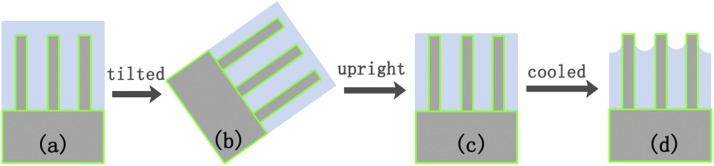
Schematic of selective removal of the oxide layer on the rod head to form patterned protruded seed. a) Immerse the substrate into a molten wax liquid. b) Take out the substrate and run away the surplus liquid by inclined placement. c) Flatten the liquid among the rods by upright placement. d) Cool down the filled wax liquid, and it becomes solid and contracted, thus showing up the rod heads for selective removal of the oxide layer by wet etch.

Among the procedure of the prototype, selective removal of the oxide layer on the rod head is vital for the successful fabrication of patterned protruded seeds. Figure 2 shows the schematic procedure of selective removal of oxide layer on the rod head by using wax as the mask. Firstly, after the thermal oxidization, the substrate is immersed into a molten wax liquid which pushes away the air between the rods and completely fills the space among the rods. Secondly, the substrate is taken out from the wax liquid and kept inclined to run away the surplus liquid on the top of the rods, at the same time, the filled liquid will be retained because of its adhesiveness and the blocking effect of the rods. Thirdly, the substrate is placed horizontally and the filled wax liquid will get self-flattened. Finally, the heating system is shut off and the filled wax liquid gets cooled to room temperature and becomes solid, and the wax will contract a little because of the phase transition from liquid to solid, thus showing up the rod heads for selective removal of the oxide layer by wet etch. By the way, due to the adhesiveness of the wax liquid, there is always a thin wax layer sticky on the rod heads when the wax liquid becomes solid and shrinks. Hence, before the wet etch, the substrate should be put into a slightly soluble organic solvent of wax to get rid of its thin layer on the rod heads. The selective wet etch was here performed by buffered oxide etcher (BOE), and the etching duration was used to control the etched depth down to the rods, i.e., the length of the seeds.

Specifically, the fabrication of protruded seeds took the following procedure: i) kept the substrate with patterned rods at 1000°C under pure oxygen atmosphere, and got 300-500nm thick oxide layer on the rods and the substrate; ii) immersed the substrate into a wax liquid in an oven at 60°C, and kept it there for 10min to let air come off from the space between the rods, so got the rod array completely filled with liquid wax; iii) took the substrate out of the wax liquid and positioned it in the oven by the incline of 60 degree for at least 30min, thus let the surplus wax automatically run away from the top of the rod array; iv) flattened the filled wax liquid between rods by horizontally placement in the oven for 1 hour; v) shut down the oven and the substrate cooled down to room temperature, and the filled wax liquid became solid; vi) got rid of the wax thin layer on the rod heads by acetone bath for 5min; vii) selectively removed the oxide layer on the rod heads by BOE solution etching for 4min under the masking of the filled wax solid; viii) completely removed the filled wax solid by the dissolution of tetrahydrofuran.

### C. Epitaxial growth of silicon films

When the substrate with patterned protruded seeds was ready, the epitaxial growth of silicon film was performed at 1050°C under atmosphere pressure in a chemical vapor deposition system with cold walls. SiHCl_3_ gas (Arkonic Gases & Chemicals Inc., 99.999%) was used as the silicon source material with H_2_ (Airgas, 99.9999%) as the carrier gas. The HCl gas (APK gas, 99.999%.) was used to inhibit silicon nucleation on the SiO_2_ mask layer, i.e., to enhance the selective growth on the seeds.^21^ After the seeds grew up and merged into a continuous film, the HCl gas was shut off, and the growth of film went on till the desired thickness was obtained. The typical growth duration was 20min.

### D. Morphology characterization

The morphologies of the samples were characterized by the scanning electronic microscope (Hitachi S-3400N) and the optical microscope (OM, Keyence, VK-9710).

## III. RESULTS AND DISCUSSION

**FIG. 3.**
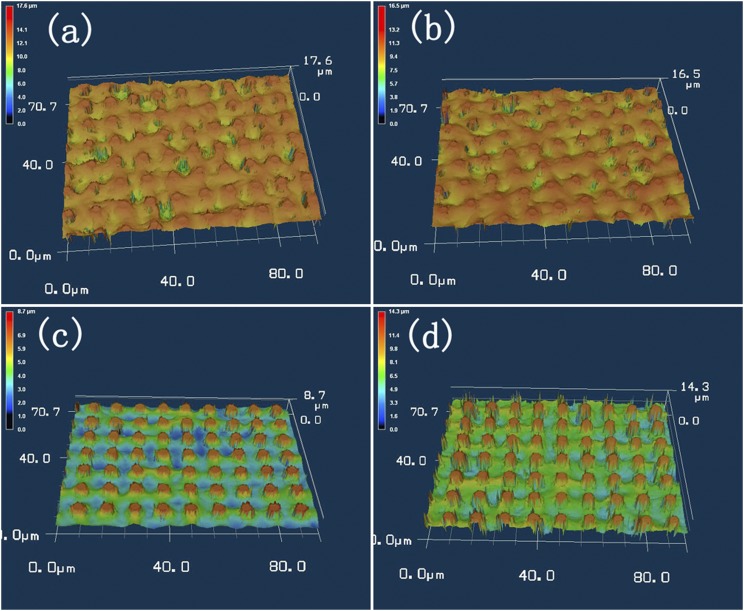
Optical-microscopic images of the substrates with the space between the rods filled by wax solid with different duration of inclined placement when the wax was still liquid. (a) 30min; (b) 1 hour; (c) 5 days; (d) 20 days.

Figure 3 shows the optical-microscopic morphologic images of the substrates when the space between the rods was filled by wax solid. The duration of inclined placement when the wax was still liquid is different, 30min, 1hour, 5 days, and 20 days, respectively for Figure 3(a) to Figure 3 (d). Obviously, in all the four cases, when the wax liquid was cooled, it didn’t bring a flat solid surface between the rods, and there were also some random pits in the filled surface, but there was always solid wax sticky around the rods; and the rods shot out of the solid wax, meanwhile, the amount of solid wax held between the rods decreased with the duration of inclined placement. Additionally, the filled amount of wax didn’t show much difference when the inclined placement duration was 30min and 1hour, same when 5 days and 20 days. It is not hard to understand that because of the blocking effect of the rods, viscous wax liquid will run very slowly across the rods, thus the difference was hardly discernible between Figure 3 (a) and Figure 3 (b); at the same time, because of the adhesiveness of the wax liquid, no more wax liquid can run away from around the rods when the duration of inclined placement is more than days, so the filling appeared fairly same in Figure 3 (c) and Figure 3 (d). As for the unevenness and pits of the filled wax solid, we thought the reason was that the used wax was an alkane mixture with short carbon chains, these alkane molecules didn’t tightly tangle and bind each other like polymer molecules, and they preferred to agglomerate around the rods when the wax liquid cooled down, then the pits turned up if some spots without enough liquid. And we also found that the bulky film of wax on a glass slide showed a rugged surface.

**FIG. 4.**
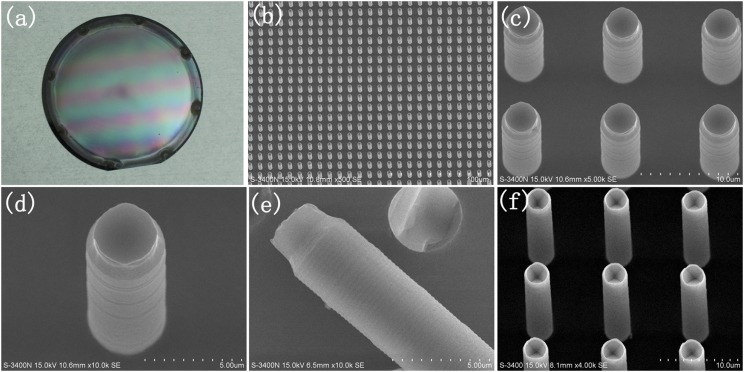
Images of the protruded seeds. (a) Photo-picture of a 4″ Cz-Si wafer all covered with protruded seeds. (b) Scanning electronic microscopy (SEM) image of the patterned protruded seeds in a large area. (c) Zoomed-in SEM image on several seeds. (d) Magnified SEM image on a single seed. (e) Magnified SEM image on a broken seed. (f) SEM image of seeds etched by KOH solution.

The obtained protruded seeds are displayed in Figure 4. Across the whole 4″ wafer substrate, the seeds are patterned with a uniform size exhibiting the excellence of wax as the filled mask for fabrication of wafer-sized protruded seeds. In particularly, the exposed silicon core has same length for each rod, this indicates that the oxide layer was just selectively removed on the rod head and controlled by its part out of the wax solid. When KOH solution was used to etch the exposed silicon head, it was found inverted pyramidal pits on each rod head, indicating the oxide layer was completely removed on the rod head (shown in Figure 4f). Evidently, although the filled wax solid didn’t show a flat surface and was distributed many random pits, these phenomena didn’t prevent the wax from functioning as a good selective wet-etch mask for the rods. In consideration of the hydrophobic properties of solid wax surface, we thought that when the etchant solution ran past and covered the substrate, the air in the pits was trapped and also worked as a mask for the substrate below it; and the etching just happened to the outer part of the rod on the wax solid; moreover, the length of the outer part of the rod was inherently depended on the viscosity of the wax liquid, this was same to each rod on a single substrate, therefore, the length of exposed silicon core for each rod was same.

According to our experiments, there are some virtues for wax used as a filled mask: i) Owing to the fluidity of molten wax liquid, its filling is uniform no matter what size of the substrate is. ii) Due to the fluidity, the surplus wax liquid is easy to dislodge by adequately inclined placement, keeping the filling right happen in the empties between the rods, and the uniformity of the liquid filling is recoverable by horizontal placement of the substrate. iii) The solid of wax is soft at room temperature, and the softness will relieve the stress of phase transition from liquid to solid, thus keep the wax sticky to the rods when the wax liquid becomes solid, and make the filled wax be a good etch resist for the rods. iv) There is no course of solvent evaporating and solution condensing, which keeps the filled solid tightly seamed with the rods (when using the photo-resist as the filling matter, there were gaps between the rod and filled photo-resist, shown in the supplementary documents).

**FIG. 5.**
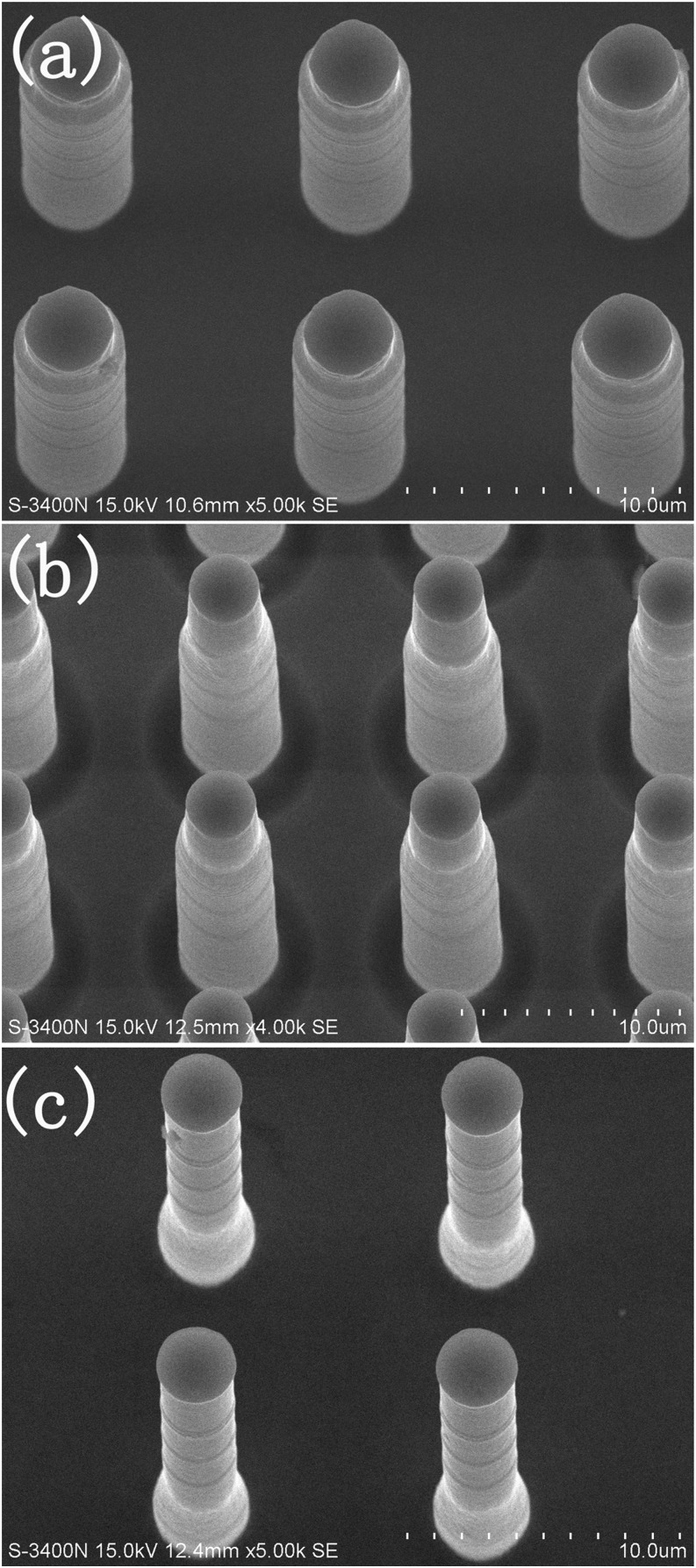
SEM images of the protruded seeds with controlled length by different wet-etching time in BOE solution. (a) 4min. (b) 8min. (c) 20min.

Figure 5 presents the manipulation of the length of the seeds by wet etch time. In order to completely remove the oxide layer on the outer part of the rod on the wax solid, the wet-etching was often performed for 4min (Figure 5a). As the etching time was extended, the etching would go down along the rods through the wax solid, then showing longer seeds (Figure 5b and 5c). The controllability of seed length further proved the superior masking effect of the filled wax.

**FIG. 6.**
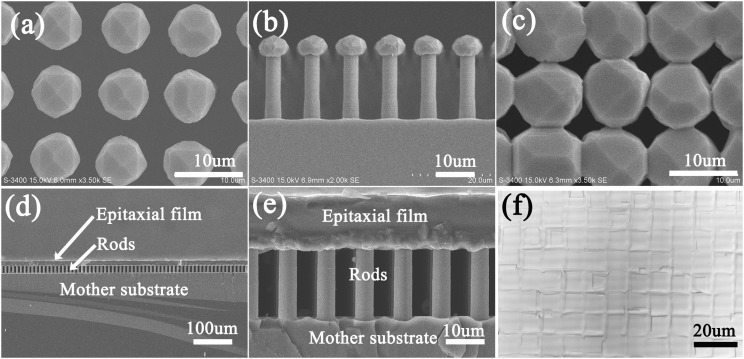
Images revealing the growth of silicon films using the prepared patterned protruded seeds. (a) Top view of the growing seeds. (b) Side view of the growing seeds. (c) Top view of the just immerged neighboring seeds. (d) Cross sectional view of the grown continuous film with the substrate. (e) Magnified cross sectional view of the grown film with the substrate. (f) Top view of the grown film.

The selectively epitaxial growth of silicon film was shown in Figure 6. At the first stage, the seeds were grown into polyhedral crystal particles, showing the nature of crystallographic anisotropic growth. As the growth evolved, the seeds grew larger and the neighboring seeds were merged (Figure 6c), and eventually became an integral film without any apertures (Figure 6d, Figure 6e, and Figure 6f), but there were square-like lightly texturized surface structures on the grown film which had the same periodicity as the original rod pattern. At the same time, no particles were observed on the surfaces of rods and the mother substrate (Figure 6b and Figure 6e), indicating that there was no growth started on the oxide mask layer. Since the nucleation and growth rate is much faster on silicon surface than on SiO_2_ surface under SiHCl_3_+HCl+H_2_ reactants system, if nuclei existed on the oxide layer, they would grow into big observable particles dotted the rods and substrate.^22^ On the other hand, the cleanness of the rods and substrate also manifested that the trapping effect of the rod jungle for reactant species didn’t significantly improve the hetero nucleation on the oxide layer. The original empties between the rods was preserved intact, leaving channels for further investigation in future on the lift-off of the grown films, reuse of the seeds, and surface engineering on both sides of the grown films when still on the mother substrate. The observed preferred growth of silicon on the protruded seeds, the obtained voidless film from discretely distributed seeds, and the as-formed structured hollows between rods verified our prototype with the potential to fabricate of transferrable films/wafers by reusable substrates.

**FIG. 7.**
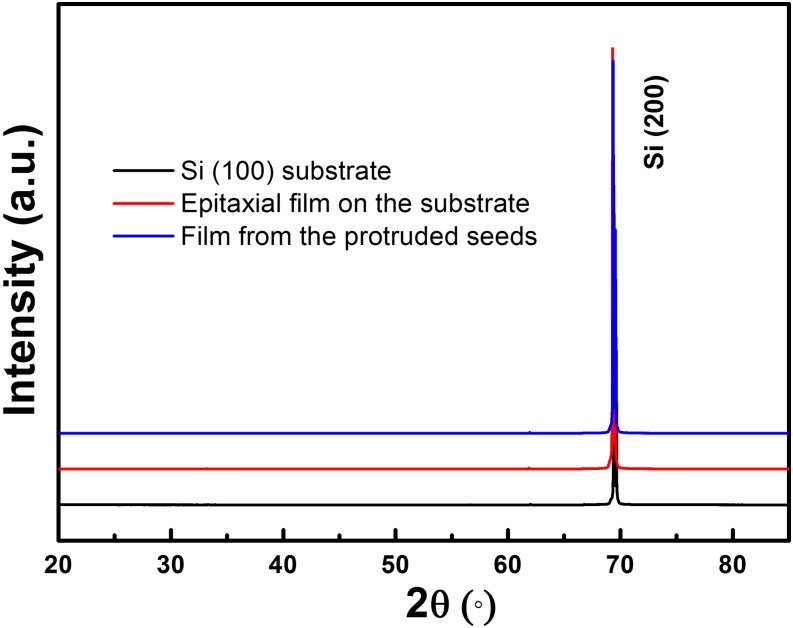
XRD spectra of the grown film from the patterned protruded seeds, of the epitaxial film directly on the Si (100) substrate, and of the Si (100) substrate.

The crystal quality of the grown films have been characterized by X-ray diffraction (XRD) spectra, as shown in Figure 7, indicating that the grown film from the patterned protruded seeds has the almost same crystalline quality as the epitaxial one directly on the Si(100) substrate and the substrate itself. This confirmed the fact that complete and continuous film with the same crystal quality as the mother substrate could be grown from the discretely distributed protruded seeds.

## IV. CONCLUSION

In summary, we demonstrated the prototype using discrete protruded seeds to grow voidless and continuous silicon film, and the method using wax as the filled mask to selectively remove the oxide layer on the heads of the patterned rods has been developed for fabrication of protruded seeds. The method of fabricating filled mask for patterned rod array using liquid wax is greatly applicable to wafer-sized preparation of protruded seeds now that the fluidity of the wax liquid can facilitate uniformly filling all over any sized area. Moreover, the length of seeds along the rods was controllable by wet-etching time. The selective epitaxial growth of silicon films was performed on the ready patterned protruded seeds, and voidless films were obtained while the original empties between the rods were intact. It has to be pointed out that the primary target of our prototype is to build uniformly structured hollows between the grown film and the mother substrate to realize the functions such as transferability of the grown film, accessibility of gases or solutions to the below surface of the film when it still staying on the mother substrate, and reusability of the seeds. According to the experiment results, the structured hollows were readily obtained by our prototype. Although much effort should be further paid to the investigation on lifting off the grown film and reuse of the seeds, and even the prototype should be further upgraded, the principle from our prototype did offer a potential solution to kerfless wafers and flexible crystals.

## SUPPLEMENTARY MATERIAL

See supplementary material for experiments about acetone soaking to get rid of wax thin layer on the rod head, photo-resist used as a filling matter between rods by spin-coating.
